# Transcriptomic and Proteomic Analyses of a *Wolbachia*-Free Filarial Parasite Provide Evidence of Trans-Kingdom Horizontal Gene Transfer

**DOI:** 10.1371/journal.pone.0045777

**Published:** 2012-09-26

**Authors:** Samantha N. McNulty, Sahar Abubucker, Gabriel M. Simon, Makedonka Mitreva, Nathan P. McNulty, Kerstin Fischer, Kurt C. Curtis, Norbert W. Brattig, Gary J. Weil, Peter U. Fischer

**Affiliations:** 1 Infectious Diseases Division, Department of Medicine, Washington University School of Medicine, St. Louis, Missouri, United States of America; 2 The Genome Institute, Washington University School of Medicine, St. Louis, Missouri, United States of America; 3 Center for Genome Sciences and Systems Biology, Washington University School of Medicine, St. Louis, Missouri, United States of America; 4 Department of Genetics, Washington University School of Medicine, St. Louis, Missouri, United States of America; 5 Bernhard Nocht Institute for Tropical Medicine, Hamburg, Germany; Hospital for Sick Children, Canada

## Abstract

Most filarial parasites in the subfamilies Onchocercinae and Dirofilariinae depend on *Wolbachia* endobacteria to successfully carry out their life cycle. Recently published data indicate that the few *Wolbachia*-free species in these subfamilies were infected in the distant past and have subsequently shed their endosymbionts. We used an integrated transcriptomic and proteomic analysis of *Onchocerca flexuosa* to explore the molecular mechanisms that allow worms of this species to survive without a bacterial partner. Roche/454 sequencing of the adult transcriptome produced 16,814 isogroup and 47,252 singleton sequences that are estimated to represent approximately 41% of the complete gene set. Sequences similar to 97 *Wolbachia* genes were identified from the transcriptome, some of which appear on the same transcripts as sequences similar to nematode genes. Computationally predicted peptides, including those with similarity to *Wolbachia* proteins, were classified at the domain and pathway levels in order to assess the metabolic capabilities of *O. flexuosa* and compare against the *Wolbachia*-dependent model filaria, *Brugia malayi*. Transcript data further facilitated a shotgun proteomic analysis of *O. flexuosa* adult worm lysate, resulting in the identification of 1,803 proteins. Three of the peptides detected by mass spectroscopy map to two ABC transport-related proteins from *Wolbachia*. Antibodies raised to one of the *Wolbachia*-like peptides labeled a single 38 kDa band on Western blots of *O. flexuosa* lysate and stained specific worm tissues by immunohistology. Future studies will be required to determine the exact functions of *Wolbachia*-like peptides and proteins in *O. flexuosa* and to assess their roles in worm biology.

## Introduction

Filarial nematodes are a family of medically and economically significant parasites that infect all classes of vertebrates except fish [1]. Eight filarial species parasitize humans, but most morbidity is caused by *Wuchereria bancrofti*, *Brugia malayi*, and *Onchocerca volvulus*. Global efforts are underway to eliminate transmission of these three organisms because of their significant impact on human health [2], [3]. Unfortunately, the drugs employed in these efforts have limited activity against adult filarial worms [4]. More effective therapies would significantly strengthen disease elimination efforts and improve prospects for a world without disabling filarial diseases like elephantiasis and river blindness.

Most filarial parasites of humans and domestic animals contain a bacterial endosymbiont (*Wolbachia pipientis*) that is required for development and reproduction [5]. Antibiotic treatments that clear *Wolbachia* cause stunted growth, infertility, and eventual death of adult filarial worms [6], [7]. This suggests that the *Wolbachia*-mediated biochemical pathways or processes required by filarial nematodes could serve as drug targets [8]. Although several hypotheses have been suggested regarding bacterial contributions to filarial biology [9], the molecular mechanisms that underlie this fascinating relationship are poorly understood. Therefore, the best pathways or processes to target have not been identified.

Most medically and economically significant filarial nematodes belong to two subfamilies, the Onchocercinae and the Dirofilariinae [10]. Surveys suggest that the majority of these species are *Wolbachia*-dependent [11], [12], [13]. General agreement between the phylogenies of *Wolbachia* and filarial nematodes implies that the initial infection occurred in an ancestor of the Onchocercinae and Dirofilariinae and that the worms and bacteria co-evolved thereafter [12]. The *Wolbachia*-free species in these subfamilies, which are scattered throughout the lineage, most likely arose through secondary loss of the endosymbiont [12]. Our recent genomic surveys of two distantly related *Wolbachia*-free species, *Acanthocheilonema viteae* and *Onchocerca flexuosa*, revealed evidence of horizontal gene transfer (HGT) from *Wolbachia*, supporting the secondary loss hypothesis [14].

Clearly, *Wolbachia*-independent worms must possess some “enhanced” biochemistry in relation to their *Wolbachia*-dependent counterparts. They must have developed a mechanism of synthesizing or scavenging critical resources that *Wolbachia*-dependent species obtain from their endobacterial partner. In some cases, *Wolbachia*-free worms may have evolved their own compensatory mechanisms. However, it is also possible that they acquired essential *Wolbachia* genes or biochemical pathways from a former endosymbiont via HGT given that they were associated with *Wolbachia* in the past. Evidence for either possibility should be present in the genomes of *Wolbachia*-free filarial species. The identification of compensatory or *Wolbachia*-like genes/pathways may provide clues as to the nature of the interactions between *Wolbachia* and filarial nematodes and suggest targets for drugs aimed at disrupting this critical relationship in *Wolbachia*-dependent human and animal pathogens.

In this study, we used an integrated transcriptomic and proteomic approach to facilitate gene discovery in *O. flexuosa* (subfamily Onchocercinae), a *Wolbachia*-free parasite of European red deer [15]. Sequencing the adult transcriptome allowed us to assess this organism’s biochemical capabilities and gather a more complete inventory of transcribed *Wolbachia*-like sequences. This work confirmed and expanded our prior finding that many *Wolbachia*-like sequences are transcribed in *O. flexuosa*. A mass spectrometry analysis extended the work a step further by demonstrating the presence of *Wolbachia*-like peptides. Western blot and immunohistology results corroborated this finding and indicated that a peptide matching to a *Wolbachia* LolC protein was expressed in a tissue- and stage-specific manner in *O. flexuosa*. This suggests that *Wolbachia*-like sequences could have biological functions in *Wolbachia-*free worms and may not be merely inactive, fossilized remains of past infections.

## Results

### Transcriptome Sequencing, Assembly, and Quality Assessment

Sequencing and assembly results are outlined in Table 1. *O. flexuosa* nodules (typically containing one or a few large female worms, the developing embryos and microfilaria within the females, and potentially a smaller male worm) [15]) were collected from wild European red deer. Worm fragments were removed from nodules, and RNA was isolated, reverse transcribed and subjected to Roche/454 sequencing. Reads were assembled with publically available ESTs using a cDNA specific protocol to accommodate alternative splicing and allelic variation. Our assembly produced 16,814 isogroups (unique loci) containing 25,222 isotigs (unique transcripts or spliced variants), leaving 47,252 unassembled singletons (Table 1). 23.5% of the isogroups contained more than one isotig (mean 3.1 isotigs per isogroup for this subset), which suggests the possibility of alternate splicing (AS). For comparison, nearly 25% of *C. elegans* genes are thought to undergo some form of AS (e.g., tissue, sex or stage-specific) with an average of two isoforms per AS gene [16].

**Table 1:** Results of sequencing, assembly, and translation of the *O. flexuosa* transcriptome.

Sequencing	
Total reads	1,334,767
Average read length	350 bp
Total ESTs (Genbank)	2,124
Average EST length	443 bp
**Assembly**	
# Singletons	47,252
Average singleton length	538 bp
# Isotigs	25,222
Average isotig length	810 bp
# Isogroups	16,814
# Isogroups with one isotig	12,874
Average isotigs per alternatively spliced isogroup	3.13
**Protein Predictions**	
# Unique peptides predicted	68,402
# Isogroups with predicted peptides	16,807
# Isotigs with predicted peptides	25,205
# Singletons with predicted peptides	47,252

The conserved eukaryotic gene (CEG) mapping approach was used to estimate the completeness of our dataset [17], [18]. 101 of the 248 CEGs were identified, suggesting that 41% of all *O. flexuosa* genes are represented in our transcriptome. The *O. flexuosa* genome is expected to be similar to that of *B. malayi*, a closely related filarial nematode encoding some 14,500 to 17,800 genes [19]. Since our 16,814 isogroups only represent 41% of the complete gene set, it is highly unlikely that the 16,814 isogroups correspond to 16,814 unique genes. This is suggestive of fragmentation. Fragmentation, defined as non-overlapping isogroups derived from the same gene, was estimated to be 32%, 25% or 7%, by comparison with *B. malayi*, *C. elegans*, and the CEG dataset, respectively.

### BLAST Analyses of the *O. flexuosa* Transcriptome

59.6% of the isogroups (67.2% of isotigs) and 34.6% of the singletons were matched to known sequences in BLAST searches against various databases (Table 2). More than 95% of the isogroups and singletons had a top hit to a *B. malayi* or *C. elegans* protein in BLAST searches against the non-redundant protein database (nr). 32.0% percent of *B. malayi* protein sequences and 11.0% of *C. elegans* protein sequences had putative orthologs in the *O. flexuosa* transcriptome (Table 2), with top-hit high scoring segment pairs (HSPs) sharing an average of 80.7% and 70.0% sequence identity between *O. flexuosa* and *B. malayi* or *C. elegans*, respectively.

**Table 2:** Results of BLAST searches comparing *O. flexuosa* transcripts to various databases.

Database	DatabaseSource	Sequence search	# Isogroups	# Isotigs	# Singletons
nr	NCBI	BLASTX	6,619 (39.4%)	12,013 (47.6%)	7,278 (15.4%)
Nematoda			6,442	11,755	6,886
* Wolbachia*			62	73	36
Nematoda and *Wolbachia*			17	23	4
nt	NCBI	BLASTN	6,998 (41.6%)	12,641 (50.1%)	9,988 (21.1%)
Nematoda			6,613	12,141	8,731
* Wolbachia*			86	98	102
Nematoda and *Wolbachia*			20	22	10
Nematoda ESTs	GenBank	BLASTN	5,485 (32.6%)	9,765 (38.7%)	7,535 (15.9%)
*B. malayi* proteins	WormBase	BLASTX	6,683 (39.7%)	12,153 (48.2%)	7,702 (16.3%)
*B. malayi* genome assembly	WormBase	BLASTN	8,350 (49.7%)	14,522 (57.6%)	11,836 (25.0%)
*C. elegans* proteins	WormBase	BLASTX	2,437 (14.5%)	4,324 (17.1%)	2,385 (5.0%)
Hit to one or moredatabases			10,024 (59.6%)	16,944 (67.2%)	16,359 (34.6%)

**Figure 1:**
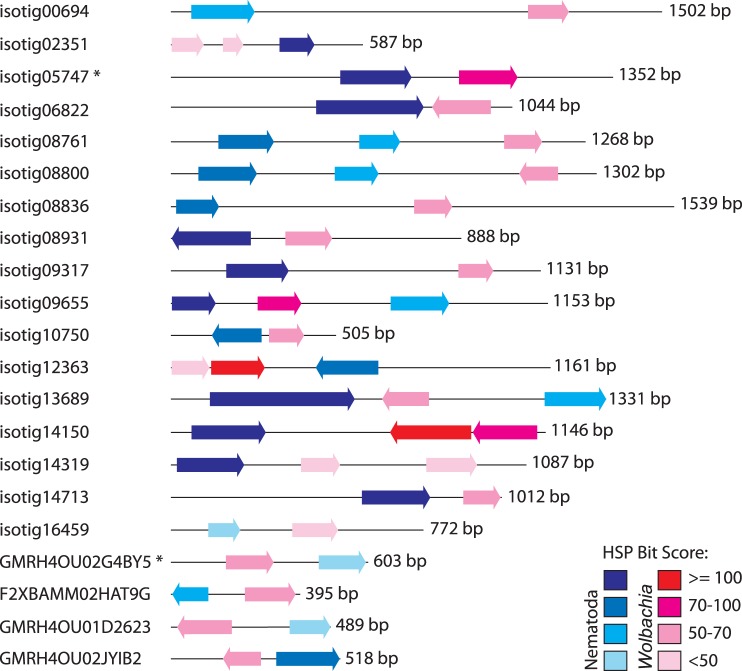
*Onchocerca flexuosa* transcripts sharing sequence identity with *Wolbachia* and nematode genes. 17 “hybrid” isogroups (23 isotigs) and four “hybrid” singletons were identified as sharing sequence identity with *Wolbachia* (red) and nematode (blue) genes in a BLAST search against the non-redundant protein database. One representative isotig from each of the 17 “hybrid” isogroups was chosen for display. Arrows indicate predicted directionality of high-scoring segment pairs, and isotigs and singletons with *Wolbachia*-like and nematode-like sequences in the same reading frame are marked with an asterisk. Color intensity was used to indicate the degree of similarity shared between a given sequence and its top BLAST hit (see key in image).

Regions of 62 isogroups (73 isotigs) and 36 singletons had top ranking hits to *Wolbachia* proteins in BLAST searches against nr (Table 2 and Table S1, see Methods for cutoff values). Altogether, these *Wolbachia*-like sequence fragments represent a total of 97 different *Wolbachia* genes (43 from filarial *Wolbachia* strains and 54 from insect *Wolbachia* strains) with diverse functions. BLAST HSPs ranged in size from 62 to 308 bp and shared an average of 70.6% sequence identity with *Wolbachia* proteins at the amino acid level. In some instances, *Wolbachia*-like sequences were present on the same transcript as nematode-like sequences (Table 2, Fig. 1). These “hybrid” transcripts were verified by PCR amplification from genomic DNA and re-sequencing in order to ensure that they did not arise from assembly errors.

**Figure 2:**
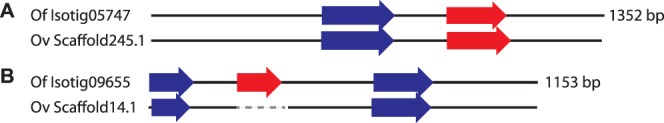
Sequence conservation between *Onchocerca flexuosa* and *O. volvulus*. *O. flexuosa* transcripts containing *Wolbachia*-like sequences were compared with the latest version of the *O. volvulus* genome assembly. Eighteen *O. flexuosa* isogroups (24 isotigs) and eight singletons with *Wolbachia*-like sequences shared at least 80% sequence identity with sequences from *O. volvulus* over 80% of the length of the *O. flexuosa* transcript. For example, *O. flexuosa* isotig05747 shares 90% sequence identity with a 1344 bp portion of *O. volvulus* scaffold245.1; both of these sequences contain a similar *Wolbachia*-like insert (A). Another nine *O. flexuosa* isogroups (9 isotigs) and one singleton matched *O. volvulus* sequences, except the latter did not contain *Wolbachia*-like inserts. For example, the sequences before and after the *Wolbachia*-like insert in *O. flexuosa* isotig09655 share 84% and 89% sequence identity with corresponding regions of *O. volvulus* scaffold14.1, respectively; however, the *Wolbachia*-like sequence present in *O. flexuosa* is not present in *O. volvulus* (B). Regions sharing sequence identity with *Wolbachia* are indicated in red while regions similar to nematode genes are indicated in blue. Arrows indicate directionality of high-scoring segment pairs. The grey dashed line represents a gap in the sequence alignment between the two species.

*O. volvulus* is a *Wolbachia*-dependent filarial nematode species in the same genus as *O. flexuosa*. Its genome has been sequenced, and it is currently being assembled and annotated. Preliminary data are publicly available, but the sequences have not yet been added to nr. The *O. flexuosa* isotigs and singletons containing *Wolbachia*-like sequences were compared against the latest version of the *O. volvulus* genome assembly. Eighteen isogroups (24 isotigs) and eight singletons matched (with greater than 80% sequence identity shared over more than 80% of the length of the *O. flexuosa* transcript) *O. volvulus* genomic sequences that contained similar *Wolbachia*-like inserts (Table S2, Fig. 2A). Nine other *O. flexuosa* isogroups (nine isotigs) and one singleton that contained *Wolbachia*-like inserts flanked by *Onchocerca* sequences matched *O. volvulus* genomic sequences that did not contain *Wolbachia*-like inserts (Table S2, Fig. 2B).

### Translation and Functional Annotation of the Transcriptome

68,402 unique peptide translations were produced from the *O. flexuosa* isotigs and singletons (Table 1). Sixty-two of these, ranging in size from 38 to 216 aa (mean 65.8±37.3 aa), had top hits to *Wolbachia* proteins by BLAST against nr. Predicted peptide sequences (including those from *Wolbachia*-like peptides), were compared to InterPro domains and KEGG orthologous (KO) groups. Collagen triple helix repeats, kinase domains and WD40 related domains were among the most commonly occurring protein domains (Table S3), while “folding, sorting and degradation”, “transcription, replication and repair”, and “signal transduction” were among the most abundantly represented pathway categories (Tables S4).

**Figure 3:**
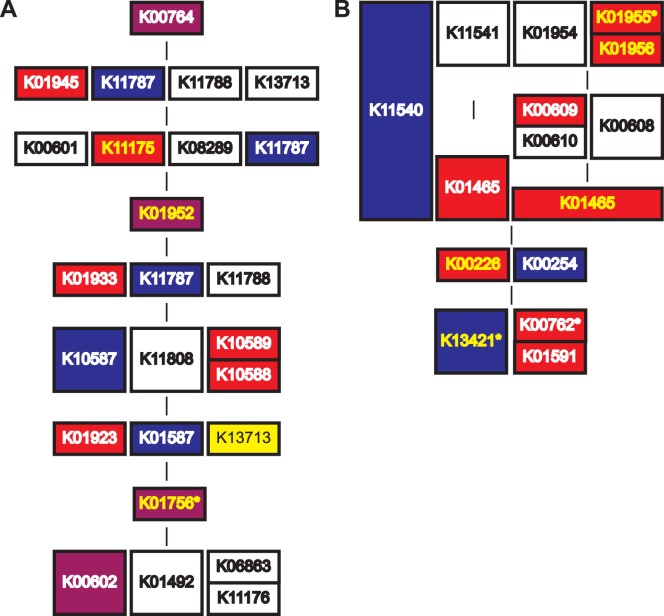
*Onchocerca flexuosa* sequences related to *de novo* purine and pyrimidine biosynthesis. KEGG pathway modules are outlined for inosine monophosphate (A) and uridine monophosphate (B) biosynthesis (module entries M00048 and M00051, respectively). Boxes representing enzymes present in nematodes (e.g., *C. elegans*), *Wolbachia*, or both are colored blue, red and purple, respectively. Enzymes sharing sequence identity with *O. flexuosa* peptide translations are highlighted in yellow. Enzymes identified from *B. malayi* are marked with an asterisk.

*O. flexuosa* peptide translations and *B. malayi* proteins assigned to KO groups were binned into pathway modules to provide a means of comparing the metabolic capabilities of these two organisms (Table S5). Genes related to the de novo synthesis of riboflavin, heme and nucleotides are missing from the nuclear genome of *B. malayi* but present in the genome of its *Wolbachia* endosymbiont, suggesting that *B. malayi* may rely on *Wolbachia* as a source of these substances [9], [19]. Table 3 highlights these four pathway modules. More sequences related to inosine monophosphate and uridine monophosphate biosynthesis (required for the *de novo* synthesis of purines and pyrimidines, respectively) were identified from *O. flexuosa* transcripts than from the nearly-complete genome of *B. malayi* (Table 3, Fig. 3), but key enzymatic steps in both pathways remain unaccounted for in *O. flexuosa*. Interestingly, some of the sequences binned into these modules are similar to enzymes from *Wolbachia* rather than enzymes from nematodes or other metazoans (Fig. 3). *Wolbachia*-like sequences could be assigned to 41 unique KO groups which were further binned into 26 pathway modules, all of which were sparsely populated (Table S6).

**Table 3:** Selected KEGG pathway modules in *B. malayi* and *O. flexuosa*. Table lists the number of module enzymes represented in each dataset.

Module	Description	Total Enzymesin Module	*B. malayi* Proteins	*O. flexuosa*	
				Peptide Translations	*Wolbachia*-Like Peptide Translations
M00048	Inosine monophosphate biosynthesis	20	1	4	2
M00051	Uridine monophosphate biosynthesis	13	3	5	3
M00121	Heme biosynthesis	9	3	3	1
M00125	Riboflavin biosynthesis	5	0	0	0

### Analysis of the *O. flexuosa* Proteome

Adult worm lysate was subjected to a shotgun proteomic analysis in order to characterize the *O. flexuosa* proteome. Using this method, proteins are identified by matching experimentally obtained mass spectra to amino acid sequences predicted from the organism’s genome. Because the *O. flexuosa* genome has not been fully sequenced, we compiled a custom comparative database for analysis of MS results. This database contained peptide translations from the *O. flexuosa* transcriptome and sequences from the phylum Nematoda and the genus *Wolbachia* (see methods). Interrogation of this database resulted in the successful characterization of 8,537 unique peptides that mapped to 2,685 database entries. Matches were further grouped into 1,803 potential proteins (Table S7). Of the 1,803 proteins, 673 matched *O. flexuosa* peptide translations, 1,077 matched sequences from other organisms, and 53 matched to both. Database entries identified by MS (available from Nematode.net [20]) were annotated in the same manner as the transcriptome. Thioredoxin folds, concanavalin A-like lectin/glucanase domains and immunoglobulin fold domains were among the most abundant protein domains (Table S3), while “Folding, sorting and degradation”, “translation”, “energy metabolism” and “carbohydrate metabolism” were among the most heavily represented KEGG pathway categories (Table S4).

**Figure 4:**
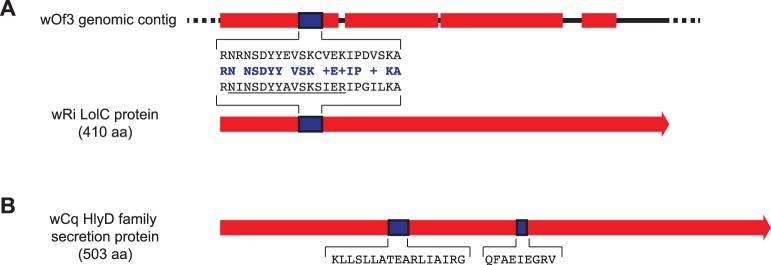
*Wolbachia*-like peptides identified by mass spectroscopy (MS). *Wolbachia* genes are shown in red with MS peptides highlighted in blue and peptide sequences shown in black text. One peptide with two charge states was mapped to a LolC protein found in several insect *Wolbachia* strains, including the *Wolbachia* endosymbiont of *Drosophila simulans* (wRi) (A), and two unique peptides were mapped to a HlyD family protein from the *Wolbachia* endosymbiont of *Culex* quinquefasciatus (wCq) (B). A region with sequence identity to the LolC gene was identified in the *O. flexuosa* genome [14], but this locus would not be capable of producing the exact peptide identified in our MS experiment (consensus shown in blue text). Polyclonal antibodies were raised against the underlined portion of the LolC peptide.

Although none of the *Wolbachia*-like peptides predicted from the *O. flexuosa* transcriptome were identified by MS, three MS peptides matched to two *Wolbachia* proteins (see MS proteins 1591 and 1637, Table S7). A 23aa peptide present in two separate charge states mapped to a lipoprotein releasing system transmembrane protein, LolC, that is present in several insect *Wolbachia* strains (Fig. 4A). A sequence similar to the *Wolbachia* LolC gene was previously identified in the *O. flexuosa* genome (see genomic fragment wOf3, [14]); however, this sequence has diverged to an extent that it would not be capable of producing the exact peptide detected by MS (Fig. 4A). Two more peptides (17 aa and 9 aa) mapped to an HlyD family secretion protein from the *Wolbachia* endosymbiont of *Culex quinquefasciatus* (Fig. 4B). Several PCR primer sets corresponding to different regions of HlyD were used in unsuccessful attempts to amplify sequences from *O. flexuosa*. Thus far, we have not identified any genomic or transcriptomic sequence capable of producing these *Wolbachia*-like peptides.

### Detection of *Wolbachia*-like Peptides in *O. flexuosa* Using Anti-peptide Antibodies

**Figure 5:**
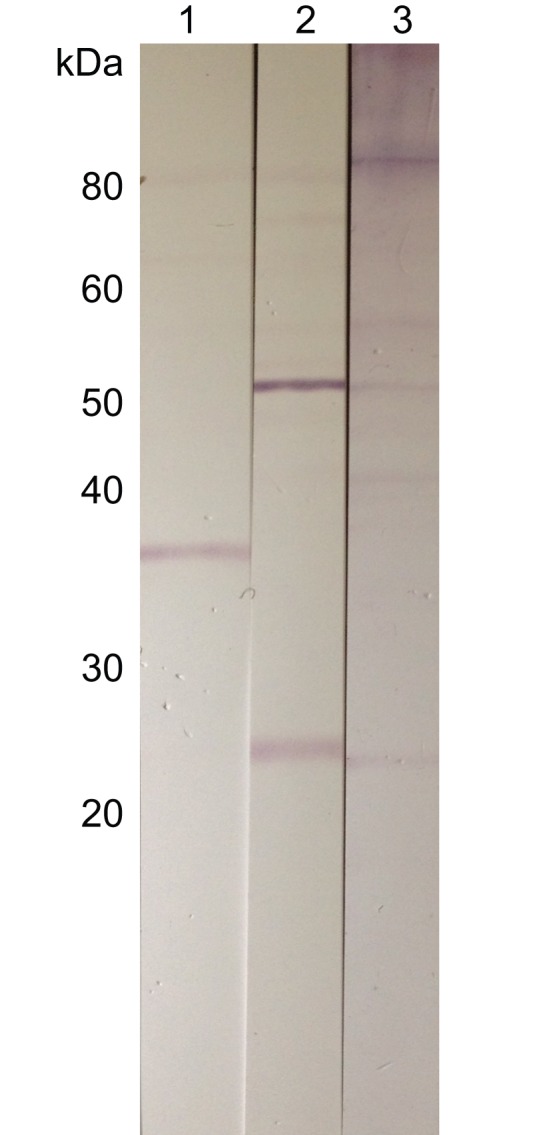
Western blots detecting the putative LolC peptide in *Onchocerca flexuosa* adult worm lysate. Affinity-purified rabbit antibodies raised against a peptide from MS protein 1591, a putative LolC homolog, bound a 38 kDa band in Western blots of *O. flexuosa* adult worm lysate (lane 1). Purified IgG from pre-immune serum (lane 2) and antibodies against the keyhole limpit hemocyanin carrier (lane 3) did not bind to proteins in this size range.

Polyclonal antibodies raised against the peptide mapping to *Wolbachia* LolC (see Fig. 4A) detected a single band at approximately 38 kDa in *O. flexuosa* adult worm lysate by Western blot (Fig. 5). Similar bands were not detected by IgG from pre-immune serum or antibodies against the keyhole limpit hemocyanin (KLH) carrier protein used in antibody synthesis. For comparison, the LolC protein from the *Wolbachia* endosymbiont of *Drosophila simulans* (accession ACN95889) is 44.9 kDa while its homolog in the *Wolbachia* endosymbiont of *B. malayi* (accession YP_198313) is 42.5 kDa.

**Figure 6:**
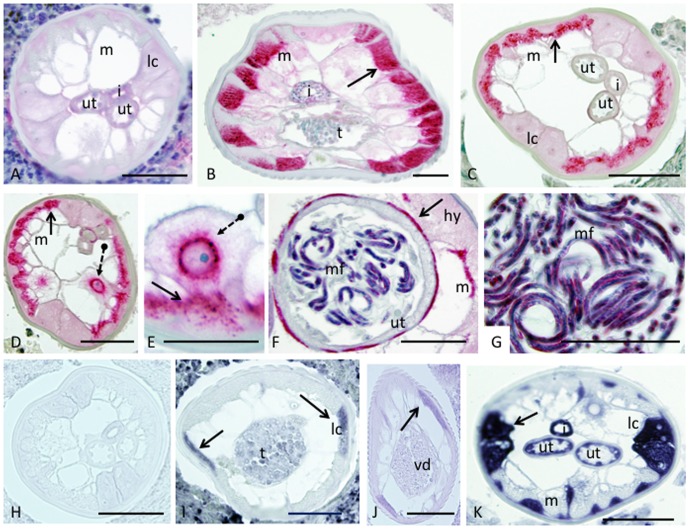
Localization of the putative LolC peptide by immunohistology and *in situ* hybridization in *Onchocerca flexuosa*. The putative LolC peptide and transcript were localized in adult worm tissues via immunohistology (A-G) and *in situ* hybridization (H-K), respectively. Negative control antibodies against keyhole limpit hemocyanin (carrier protein) showed no specific staining in *O. flexuosa* (A). Antibodies against a peptide from MS protein 1591, a putative homolog of *Wolbachia* LolC, stained the fibrillar portions of the somatic muscles (arrow) in a cross section of an adult male worm (B). The somatic muscles (solid arrows) and excretory cell (dashed arrow) are stained in cross sections of young adult females (C, D). Magnification of the excretory cell shows intense staining of the cell membrane (dashed arrow) and more diffuse staining in adjacent muscles (solid arrow) (E). The uterine muscles of an older female (arrow) are clearly stained (F), as are the intrauterine stretched microfilaria (F, G). The LolC sense RNA probe produced no signal in *in situ* hybridizations (H), while the antisense RNA probe labeled the lateral chords (arrows) and developing sperm within the male testes (I, J). The antisense RNA probe also labeled the lateral chords (arrow), intestine, and uteri of a young adult female (K). Abbreviations: m, muscle; lc, lateral chords; i, intestine; ut, uterus; mf, microfilariae; hy, hypodermis; t, testes; vd, vas deferens. Scale bars = 25 µm.

The same antibodies were used to localize the target peptide in *O. flexuosa* worms (Fig. 6). Control antibodies to KLH did not label *O. flexuosa* (Fig. 6A), and total IgG from pre-immune serum produced the same result (not shown). In contrast, antibody to the putative LolC peptide strongly labeled fibrillar portions of the somatic muscles of adult male (Fig. 6B) and young female (Fig. 6C, D) worms. Older females have less pronounced somatic musculature and showed weaker labeling; however, the antibody labeled uterine muscles and coiled and stretched microfilariae in these worms (Figs. 6F, G). Distinct staining was also seen in the membrane of the excretory cell (Fig. 6D, E). Sequences homologous to LolC were identified from the genome of the *Wolbachia* endosymbiont of *B. malayi* and used to make RNA probes for *in situ* hybridization since corresponding sequences have not yet been identified from *O. flexuosa*. *In situ* hybridization studies indicated that the target RNA was produced in the lateral chords (Fig. 6I, J, K), the hypodermis (Fig. 6K), developing sperm (Fig. 6I), uteri (Fig. 6K) and intestine (Fig. 6K).

## Discussion

The relationship between *Wolbachia* and *Wolbachia*-dependent filarial nematodes has been a topic of keen interest in recent years since *Wolbachia* was identified as a potential anti-filarial drug target. Most research on this relationship has focused on *Wolbachia*-dependent filarial parasites of humans and domestic animals. *Wolbachia*-free species have been overlooked since most are animal parasites with no economic or medical importance. Therefore, the mechanisms allowing these species to survive without an endosymbiont remain unknown. We believe that the genetic characterization of *Wolbachia*-free filarial parasites like *O. flexuosa* may lead to a better understanding of filarial biology and the mechanisms responsible for *Wolbachia* dependence in important filarial pathogens such as *W. bancrofti*, *Dirofilaria immitis,* and *O. volvulus*.

In this study, we focused on the transcriptome rather than the genome of *O. flexuosa* as an efficient and cost-effective method for gene discovery in a *Wolbachia*-free filarial parasite. Estimates of completeness suggest that our dataset represents approximately 41% of the genes encoded in the *O. flexuosa* genome. This result was not unexpected, since RNA from nodules does not contain transcripts from all parasite stages. *O. flexuosa* nodules, and the cDNA libraries derived from them, are biased towards adult females due to the size and relative abundance of the female worms within. However, 41% is on par with the number of genes represented by unambiguously-mapped reads derived from adult female transcripts in a recent analysis of the *B. malayi* transcriptome [21]. It will be difficult to discover more genes for this species through transcriptome sequencing since these parasites are only found in wild deer and the life cycle cannot be maintained in the laboratory; therefore, it is not possible to collect material representing life cycle stages that are not well represented in nodules.

One major motivation for studying the *O. flexuosa* transcriptome was to develop a more complete inventory of transcribed *Wolbachia*-like sequences. Our previous genomic surveys indicated that *O. flexuosa* had incorporated fragments of at least 92 *Wolbachia* genes in its nuclear genome through HGT [14]. An additional 86 *Wolbachia* genes were represented in our partial transcriptome, increasing the total number of *Wolbachia* genes represented by *Wolbachia*-like sequence fragments in *O. flexuosa* to 178. Interestingly, only 11 *Wolbachia* genes were represented in both the genomic and transcriptomic datasets despite the fact that a majority of the *Wolbachia*-like sequences identified in the genome were shown to be expressed at the RNA level using qRT-PCR [14]. This disparity could be due to variation in the wild-caught samples, differences in sequencing coverage between the two studies, differences in cDNA preparation (total RNA in the previous study versus poly(A) selected RNA in the present study), or differences in sensitivity between qRT-PCR used in the prior study versus 454/Roche sequencing used in this study. No effort was made in either study to quantify the relative expression of *Wolbachia*-like gene sequences since no criteria exist to define “biologically relevant” expression levels. However, some of the isogroups found to contain *Wolbachia*-like sequences were constructed from many reads, suggesting a reasonably abundant transcript population.

It is possible that *O. flexuosa* contains many more *Wolbachia*-like sequences than those uncovered by our genomic and transcriptomic surveys, as nearly complete *Wolbachia* gene sets have been discovered in other species (e.g., *Drosophila ananassae* [22] and *Callosobruchus chinensis* [23]). Once comprehensive inventories of HGT sequences have been obtained from many species, it would be interesting to compare and contrast the *Wolbachia* genes represented in various insect and filarial genomes, especially between *Wolbachia*-dependent and independent filaria. Cataloging transferred sequences shared between *Wolbachia*-independent species but absent from *Wolbachia*-dependent species could provide clues as to which sequences, if any, could have facilitated endosymbiont loss. As one might expect, preliminarily comparisons between the *Wolbachia*-like sequences found in *O. flexuosa* and the preliminary draft of the nuclear genome of the *Wolbachia*-dependent species *Onchocerca volvulus* indicate that some *Wolbachia*-like inserts are unique to *O. flexuosa* while others are shared between the two species. Shared inserts were presumably transferred prior to separation of the two *Onchocerca* species whereas *O. flexuosa* specific inserts were presumably transferred afterwards. However, it must be noted that the *O. volvulus* genome assembly is not fully complete, and the putative transfers have not been experimentally verified. The catalogs of shared versus unique fragments will certainly grow and change as the available datasets evolve.

Despite our extensive catalog of *Wolbachia*-like sequences, we have yet to identify a full-length, uninterrupted *Wolbachia*-like gene in *O. flexuosa*. This may be due to technical issues (e.g., insufficient coverage, fragmentation, etc.). It may also be that complete genes were transferred but decayed over time in the absence of selective pressure. However, it is possible that transfers took place on the level of domains rather than complete genes. Many proteins are made up of multiple domains that each contribute to overall function, and studies have suggested that domains, rather than complete genes, might be common units of genetic transfer [24]. For example, transfers of mitochondrial DNA to the nuclear genome have generated “hybrid” proteins containing both “nuclear” and “organellar” exons [25]. In support of this hypothesis, we identified 17 isogroups and four singletons containing separate regions of similarity to *Wolbachia* and nematode genes. Most of the “hybrid” transcripts seem incapable of producing a functional product (i.e., nematode-like and *Wolbachia-*like sequences code in opposing directions or different reading frames), but some have the potential to yield a “hybrid” protein with nematode-like and *Wolbachia*-like subunits. Of course, whether the inserted *Wolbachia* domains disrupt protein function, impart novel capabilities, or have no effect at all remains to be seen.

Given that we have only sampled some 41% of the complete genome and that the transcripts are still heavily fragmented, it is difficult to make definitive statements regarding the biochemical capabilities of *O. flexuosa.* Many important biochemical pathways appear incomplete, presumably due to insufficient sequence coverage (Table S5). However, more sequences related to *de novo* purine and pyrimidine synthesis were identified from the partial transcriptome of *O. flexuosa* than the nearly complete genome of *B. malayi*. While missing from *B. malayi*, purine and pyrimidine synthesis pathways are present in other nematodes (i.e., *Caenorhabditis* species) and in the *Wolbachia* endosymbiont [9], [19], raising the possibility that *B. malayi* relies on *Wolbachia* as a source of nucleotides, particularly during times of intense demand (e.g., growth and for reproduction). Further testing will be required to determine whether the *O. flexuosa* sequences binned into these two pathway modules belong to full-length, intact, functional genes and, if so, whether nematode-like and *Wolbachia*-like enzymes could act in concert in a functional biochemical pathway. *De novo* nucleotide synthesis could contribute to *O. flexuosa*’s ability to survive without an endosymbiont, so this is an interesting possibility that deserves closer examination.

Our transcriptome data facilitated a parallel proteomic analysis of *O. flexuosa* adult worm lysate. Shotgun proteomic studies are generally restricted to organisms with sequenced genomes, as MS data are only informative if sequences from the organism of interest are available for comparison. Information from closely related species can be used when genomic data are unavailable, but only very highly conserved proteins will be identified since matches between experimentally collected mass spectra and database sequences must be exact [26], [27], [28]. Our approach of adding transcript sequences to a database of orthologous proteins from *Wolbachia* and other nematodes led to identification of 1,803 proteins in adult worm lysate. Sequence conservation between *O. flexuosa* and better-studied filarial species like *B. malayi* allowed for a high rate of cross-species matches (∼60% of matches) and greatly increased the number of proteins that we were able to identify.

The present study identified *Wolbachia*-like peptides in a *Wolbachia*-free filarial worm (*O. flexuosa*), an important step beyond what we had previously reported. Peptides mapping to *Wolbachia* HlyD and LolC were identified in adult worm lysate by MS, and the putative LolC peptide was identified by Western blot and by immunohistology using polyclonal antibodies to the peptide. Neither of these proteins has been studied in *Wolbachia*, but both LolC and HlyD are related to ABC transport in *Escherichia coli* and other bacteria [29], [30]. ABC transporters are common in both prokaryotes and eukaryotes. They interact with a diverse array of substrates, and they may play a role in drug resistance in filarial nematodes [31], [32], [33], [34]. We do not know whether the peptides mapping to LolC and HlyD exist as small inserts in larger nematode proteins or whether they represent fragments of complete *Wolbachia*-like proteins. The fact that the two peptides that mapped to HlyD are separated by 99 amino acids supports the latter hypothesis (see Fig. 4B). In any case, it is fair to say that the function(s), if any, of the putative HlyD and LolC sequences in *O. flexuosa* are unknown at this time.

Our immunohistological and *in situ* hybridization studies revealed interesting, tissue-specific patterns of expression for the putative LolC gene and peptide. To date, three *Wolbachia* like transcripts (homologs of 2-methylthioadenine synthase and DNA polymerase I previously described in [14] and LolC described in Fig. 6 of this paper) have been localized, and all three exhibit similar expression patterns. While various tissues appear to be capable of producing *Wolbachia*-like transcripts, strong transcription signals were observed in tissues that harbor *Wolbachia* in infected species (e.g., lateral chords and reproductive organs) (see [35] for localization of *Wolbachia* in *B. malayi*; see [14] and Fig. 6 in this paper for localization of *Wolbachia*-like transcripts in *O. flexuosa*). However, immunohistology studies showed that the putative LolC peptide was mainly present in fibrillar portions of muscle. This does not correspond to the *in situ* hybridization results. mRNAs and their corresponding proteins are generally found in the same tissues, but there are precedents for disparate localization of transcripts and proteins derived from the same gene. For example, the filarial gene *shp-1* is expressed in the uterine epithelium of female worms, while the protein localized to the sheath of microfilariae [36], [37]. Our results suggest that tissues known to harbor *Wolbachia* in infected worms are at least partially responsible for the production of *Wolbachia*-like products in *Wolbachia*-free worms, and that these proteins are transported to other tissues after they are produced.

We hypothesize that technical issues limited our ability to detect more *Wolbachia*-like proteins. These hindrances include the suboptimal comparative database (discussed previously), the complexity of our analyzed sample, and the relative paucity of *Wolbachia*-like proteins compared to other proteins in the sample. Detection of *Wolbachia* proteins from both infected and uninfected filarial species might benefit from a more directed approach, as certain tissues (i.e. lateral chords, ovaries, developing sperm) or lifecycle stages (i.e. adolescent worms) may express these genes to a higher degree than others (see [14], [35] and Fig. 6). Analyses of isolated tissues might lead to a higher identification rates for *Wolbachia*-like proteins and peptides in tissues from both infected and uninfected filarial species.

Thus far, our studies have provided compelling evidence that *Wolbachia*-like sequences are present in the genome of *O. flexuosa*, and that they are expressed at the RNA and protein levels in a tissue- and stage-specific manner. Although the preservation, expression, and regulation of these sequences suggest that they may play important roles in filarial biology, expression does not guarantee utility. It is possible that *Wolbachia* sequences could insert themselves into existing *O. flexuosa* proteins without imparting new functions, and it is possible that even full-length, abundantly expressed *Wolbachia*-like proteins could be useless in the context of the worm interactome. Clearly, additional studies will be needed to determine the significance of *Wolbachia*-like sequences in *Wolbachia*-free filarial nematodes and to determine whether they are capable of supporting the independent, *Wolbachia*-free lifestyle of these species.

## Materials and Methods

### Parasite Material

Nodules containing *O. flexuosa* worms were taken from the skins of freshly-shot European red deer (*Cervus elaphus*) in northern Germany (Schleswig-Holstein) as previously described [15]. Several nodules were dissected immediately after excision from the deer, and the worm fragments pooled and stored in TRIzol (Invitrogen, Carlsbad, CA, USA) at −80°C prior to RNA isolation. Some nodules were frozen whole at −80°C and dissected at a later time to obtain worms for the proteomic study while others were fixed in 4% buffered formalin, embedded in paraffin and sectioned according to standard histological technique for use in *in situ* hybridization or immunohistology.

Adult *B. malayi* were obtained from experimentally infected Mongolian gerbils as previously described [38]. Animals were handled in accordance with guidelines defined by the Animal Welfare Act, the Guide for the Care and Use of Laboratory Animals, and the Division of Comparative Medicine, Washington University School of Medicine. Animal work was approved under WUSM Institutional Animal Care and Use Protocol 20110292. Worms were stored at −80°C prior to use.

### RNA Isolation, Library Construction and 454 Sequencing

Total RNA was isolated from *O. flexuosa* worm fragments, DNase treated and tested for DNA contamination as previously described [14]. RNA yield and integrity were assessed using a NanoDrop ND-1000 UV-VIS spectrophotometer (NanoDrop Technologies, Wilmington, DE) and a Bioanalyzer 2100 (Agilent Technologies, Cedar Creek, TX), respectively. Full-length cDNA was generated from 1.0 µg total RNA using an optimized 27-cycle protocol with the Accuscript HF Reverse Transcriptase Kit (Agilent) and SMART primers (Invitrogen). The resulting cDNA library was normalized with the Trimmer kit (Evrogen, San Diego, CA) and amplified over 14 cycles using SMART primers (Invitrogen) and Clontech Advantage-HF 2 polymerase (Clontech/Takara Bio, CA). 300–800 bp fragments were selected using AMPure paramagnetic beads (Agencourt, Beckman Coulter Genomics, Beverly, MA) following removal of 3′ and 5′ adaptors by restriction digest and processed using the Titanium General Library Kit (Roche, Branford, CT). Sequencing was performed on the Genome Sequencer 454 Titanium instrument using the GS FLX Titanium Sequencing Kit (Roche) according to standard protocol [39].

### Assembly and Translation of Transcript Sequences

Raw 454 reads were trimmed for adapters and low complexity regions using SeqClean (http://www.tigr.org/tdb/tgi/software) and screened against UniVec (http://www.ncbi.nlm.nih.gov/VecScreen/UniVec.html) and genome sequences from *Bos taurus* (the closest sequenced relative of the deer host) and *Homo sapiens*. The Newbler v2.5 assembler (Roche) was used to assemble clean reads and 2,124 Genbank ESTs (September 2010) using the following parameters “-cdna –ml 100–mi 95–icl –het”. According to this protocol, contiguous sequences (contigs) are clustered into “isogroups” that encompass all of the sequences related to a given locus. Within each isogroup, the contigs are tiled into “isotigs” meant to represent individual transcripts or splice variants. Following assembly, any isotig or singleton less than 200 bp, with more than 10% ambiguous bases, or with a top BLAST hit to a human or ruminant nucleotide or protein sequence was excluded from analysis. Isotig sequences were deposited in the GenBank transcriptome shotgun assembly database under BioProject number 62565 (accession numbers JI459010-JI484230). All sequences used in subsequent analyses, including singletons, are available at Nematode.net [20].

Peptide translations were obtained from isotigs and singletons using Prot4EST [40], [41].

### Core Eukaryotic Gene Mapping and Assessment of Fragmentation

The coverage of the *O. flexuosa* transcriptome was estimated by profile searching isotigs and singletons against core eukaryotic genes [17], [18] using HMMer [42]. A custom perl script was used to calculate the fragmentation rate based on WU-BLAST (http://blast.wustl.edu) BLASTX alignments to *B. malayi* proteins (brugpep.WS225.fa), *C. elegans* proteins (wormpep.WS21.fa), and the core eukaryotic gene set since the *O. flexuosa* genome is unavailable. The presence of multiple, non-overlapping matches to a given *B. malayi* or *C. elegans* protein was taken as evidence for fragmentation.

### BLAST Analyses

All BLAST searches were performed using blastall version 2.2.22 with the following cutoff values: e-values less than 1e-05, bit scores greater than 35 bits, and percent identity greater than 55%. Databases queried included: non-redundant protein and non-redundant nucleotide databases (downloaded 5/27/2010), all Genbank ESTs related to the Nematoda (downloaded 1/6/2011), WormBase *B. malayi* genome assembly (b_malayi.WS221.dna.fa), WormBase *B. malayi* predicted proteins (brugpep.WS221.fa), WormBase *C. elegans* predicted proteins (wormpep.WS215.fa), the *O. volvulus* genome assembly (available from The Wellcome Trust Sanger Institute, ftp://ftp.sanger.ac.uk/pub/pathogens/Onchocerca/volvulus/, last modified 2/27/2012), and the KEGG protein database (version 57). A custom perl script was used to extract the top hit from each region of the query before parsing BLAST results.

### Mass Spectroscopy

*O. flexuosa* worms were dissected from frozen nodules, pulverized in liquid nitrogen, dissolved in 50 mM Tris pH 8.0 with cOmplete protease inhibitor cocktail (Roche), and sonicated on ice. Two nodules were prepared in this manner to provide biological replicates. Half of each sonicated sample was pooled and subjected to centrifugation at 20,000 g for 90 minutes to separate soluble from insoluble proteins. This resulted in four separate samples (nodule 1, nodule 2, mixed soluble fraction, and mixed insoluble fraction) that were treated in the same manner. Protein concentration was determined by the DC Protein Assay (Bio Rad, Hercules, CA). Proteins were denatured with 8 M urea, reduced with 5 mM tris (2-carboxyethyl) phosphine (TCEP), and alkylated with iodoacetamide, prior to digestion with sequencing-grade trypsin (1∶50 trypsin:protein ratio).

Peptides from the four samples were separated using multidimensional chromatography (MudPIT) and analyzed by tandem MS as previously described [43]. Briefly, 50 µg of peptides were pressure-loaded onto a biphasic capillary column packed with strong cation exchange (SCX) and C18 resins, and fractionated by a combination of ion exchange and reverse phase chromatography. Peptides were eluted from SCX resin with six injections of increasing concentrations of ammonium acetate, followed by 120 minute organic gradients to elute peptides from the C18 resin directly into an LTQ Orbitrap Discovery hybrid mass spectrometer (Thermo Scientific, West Palm Beach, FL). MS1 spectra were acquired in the orbitrap (FTMS) with a resolution of 30,000 followed by seven data-dependent MS2 spectra in the ion trap (ITMS) at low resolution. Data were stored in Thermo RAW format, and converted to MS2 format using RawXtract 1.9.9.2 [44].

MS2 spectra were searched using the ProLuCID algorithm [45] against a combined database containing 238,403 sequences derived from *O. flexuosa* peptide translations, all GenBank protein entries from the phylum Nematoda and genus *Wolbachia* (downloaded February 2010), *WormBase* protein sequences from *B. malayi* (brugpep.WS221.fa) and *C. elegans* (wormpep.WS215.fa). Reversed “decoy” versions of each entry were also included to allow for estimation of false-discovery rates [46]. Peptide spectral matches were filtered using DTASelect 2.0 [47] resulting in a peptide false-positive rate of 1.2%.

### Functional Annotation of Predicted and Experimentally Verified Proteins

InterProScan version 4.5 was used to identify conserved domains from peptide translations and database protein sequences with matches to MS peptides [48]. Sequences were assigned to KEGG orthologous groups by comparison with the KEGG protein database [49]. Custom perl scripts were used to bin orthologous groups into broad categories and KEGG modules.

### Antibody Production

A 15 amino acid portion of MS protein 1591 (see Table S7, Fig. 4A) was selected based on inferred immunogenic properties (i.e. predicted secondary structure and hydrophobicity). Polyclonal antisera were raised against the synthetic peptide coupled to KLH in two rabbits (LifeTein LLC, South Plainfield, NJ). Antibodies were affinity purified with the original peptide and tested by ELISA prior to use (LifeTein LLC). Polyclonal antisera against the KHL carrier protein were raised, purified, and tested in the same manner. Total IgG were purified from rabbit pre-immune sera using the Protein A Agarose Kit (KPL, Gaithersburg, MD) for use as negative controls.

### Western Blots

Adult *O. flexuosa* were dissected from nodules and homogenized on ice in RIPA buffer (G Biosciences, Maryland Heights, MO) and ProteaseArrest (G Biosciences) in a one mL mini homogenizer (GPE Scientific Limited, Leighton Buzzard, UK). Homogenate was spun at 19,000 g for 15 minutes to pellet debris, and the protein concentration of the supernatant was determined using the BCA method (Pierce, Rockford, IL). *O. flexuosa* protein was subjected to SDS-PAGE using a 4–12% reducing gel (NuPAGE BisTris Mini Gel, Invitrogen) according to the manufacturer’s suggested protocol. Separated proteins were transferred to a nitrocellulose membrane (Invitrogen). The membrane was blocked overnight at 4°C in blocking buffer (0.5% Tween, 5% nonfat dry milk in 1×PBS), washed in 1×PBS with 0.5% Tween (PBS/T), and cut into strips for blotting. Antibodies were diluted in PBS/T to final concentrations of 4.4 µg/mL, 15 µg/mL and 50 µg/mL for anti-MS1591, purified pre-immune IgG and anti-KLH, respectively. Blot strips were incubated with primary antibody dilutions overnight at 4°C and washed with PBS/T at room temperature. Strips were then incubated with anti-rabbit IgG(Fc) AP conjugate (1∶3,500 in PBS/T) (Promega, Sunnyvale, CA) for 1 h at 37°C, washed with PBS/T at room temperature and developed using NBT/BCIP substrate (Promega). Substrate reaction was stopped using 20 mM Tris-HCl pH 7.4 with 5 mM EDTA.

### *In situ* Hybridization and Immunohistochemistry

DNA was isolated from adult *B. malayi* as previously described [14] using the DNeasy Blood and Tissue Kit (Qiagen). A 369 bp portion of the LolC gene from the *Wolbachia* endosymbiont of *B. malayi* (locus Wbm0483) was amplified from *B. malayi* genomic DNA using the following primers: 5′- TCTTTCATTCTCGGCACCTCA-3′ and 5′- TGGCATTGATGGCCATATCA-3′. Biotinylated RNA probes were constructed from PCR products by *in vitro* transcription as previously described [14].

Immunohistological stainings and *in situ* hybridizations were carried out as previously described [35]. *In situ* hybridizations were performed overnight at 60°C using 1 µg/mL of RNA probe in hybridization buffer. Antibodies against MS protein 1591 (LolC) were used at 0.88 µg/mL while anti-KLH antibodies were used at 1.4 µg/mL. A total of twelve nodules were examined.

## Supporting Information

Table S1 ***Onchocerca flexuosa*** **transcripts with similarity to** ***Wolbachia*** **genes.** *O. flexuosa* isotigs and singletons were compared against the non-redundant protein database. The accession numbers, descriptions and coordinates of each BLAST hit are given for isotigs and singletons that share sequence similarity to *Wolbachia*. Note that every other isotigs was highlighted in yellow to aid in distinguishing separate isotigs from separate BLAST hits.
(XLSX)

Table S2 **Sequence conservation between** ***Onchocerca flexuosa*** **and** ***O. volvulus*** **.** Sequences are designated as conserved between the transcriptome of *O. flexuosa* and genome of *O. volvulus* if they share at least 80% ID at the nucleotide level over at least 80% of the length of the *O. flexuosa* transcript. Gaps were allowed in the alignments to account for potential introns in *O. volvulus* genomic sequences. The relevant portions of the *O. volvulus* genomic scaffolds are included in this table for convenience.
(XLSX)

Table S3 **Top 25 InterPro protein domains identified from** ***Onchocerca flexuosa*** **peptide translations and proteins identified by mass spectrometry (MS).** *O. flexuosa* peptide translations and protein database entries with matches to MS peptides (termed MS proteins in this table) were compared to InterPro protein domains. Translations from 3,853 isogroups and 2,121 singletons share sequence similarity with 2,804 unique InterPro domains, while 1,573 of the 1,803 protein groups identified by MS share sequence similarity with 1,516 InterPro domains.
(DOC)

Table S4 **KEGG pathway category mappings for** ***Onchocerca flexuosa*** **peptide translations and proteins identified by mass spectroscopy (MS).** Peptide translations from 5,159 isogroups and singletons were assigned to 2,049 unique KEGG orthologous (KO) groups, while 608 protein database entries with matches to MS peptides (termed MS proteins) were associated with 446 unique KO groups. The sequences were binned into broad pathway categories based on their association with these KO groups.
(DOC)

Table S5 **KEGG pathway modules represented in** ***Onchocerca flexuosa*** **and** ***Brugia malayi*** **.** After being assigned to KEGG orthologous groups, sequences from *O. flexuosa* (both peptide translations derived from the adult transcriptome and protein database entries with matches to mass spectroscopy peptides) and *B. malalyi* predicted proteins (WormBase brugpep.WS221.fa) were binned in to KEGG pathway modules in order to compare the metabolic capabilities of the two filarial species.
(DOC)

Table S6 **KEGG pathways represented by** ***Wolbachia*** **-like sequences in** ***O. flexuosa*** **.** Peptide translations sharing sequence identity with *Wolbachia* genes were assigned to KEGG orthologous groups and binned into KEGG pathway modules in order to determine what pathways/processes these sequences might be related to.
(DOCX)

Table S7 **Results of mass spectrometry analysis of** ***O. flexuosa*** **adult worm lysate.** Experimentally detected mass spectra were compared to a protein database containing peptide translations derived from the adult transcriptome of O. flexuosa and known protein sequences related to the phylum Nematoda and the genus Wolbachia (see methods for details).
(XLSX)
