# Supplementary material for: Transcriptomic and Proteomic Analyses of a Wolbachia-Free Filarial Parasite Provide Evidence of Trans-Kingdom Horizontal Gene Transfer
Source: PLoS One. 2012 Sep 26;7(9):e45777. doi: 10.1371/journal.pone.0045777 (PMC3458923; doi:10.1371/journal.pone.0045777)
Supplement: Table S3 — Top 25 InterPro protein domains identified from Onchocerca flexuosa peptide translations and proteins identified by mass spectrometry (MS). O. flexuosa peptide translations and protein database entries with matches to MS peptides (termed MS proteins in this table) were compared to InterPro protein domains. Translations from 3,853 isogroups and 2,121 singletons share sequence similarity with 2,804 unique InterPro domains, while 1,573 of the 1,803 protein groups identified by MS share sequence similarity with 1,516 InterPro domains. (DOC) [file pone.0045777.s003.doc]

**Table S3: Top 25 InterPro protein domains identified from *O. flexuosa* peptide translations and proteins identified by mass-spectroscopy (MS).**

| Peptide Translations | | | MS Proteins | | |
| --- | --- | --- | --- | --- | --- |
| InterPro Code | Description | Isogroups and Singletons | InterPro Code | Description | Proteins |
| IPR008160 | Collagen triple helix repeat | 189 | IPR012336 | Thioredoxin-like fold | 43 |
| IPR011009 | Protein kinase-like domain | 167 | IPR008985 | Concanavalin A-like lectin/glucanase | 41 |
| IPR017442 | Serine/threonine-protein kinase-like domain | 118 | IPR013098 | Immunoglobulin I-set | 40 |
| IPR000719 | Protein kinase, catalytic domain | 102 | IPR016024 | Armadillo-type fold | 39 |
| IPR011046 | WD40 repeat-like-containing domain | 67 | IPR016040 | NAD(P)-binding domain | 35 |
| IPR019781 | WD40 repeat, subgroup | 66 | IPR013783 | Immunoglobulin-like fold | 33 |
| IPR007087 | Zinc finger, C2H2-type | 63 | IPR002928 | Myosin tail | 31 |
| IPR006201 | Neurotransmitter-gated ion-channel | 61 | IPR004000 | Actin-like | 31 |
| IPR017986 | WD40-repeat-containing domain | 60 | IPR001664 | Intermediate filament protein | 30 |
| IPR015943 | WD40/YVTN repeat-like-containing domain | 59 | IPR012335 | Thioredoxin fold | 30 |
| IPR002290 | Serine/threonine-protein kinase domain | 53 | IPR001023 | Heat shock protein Hsp70 | 29 |
| IPR001680 | WD40 repeat | 52 | IPR013126 | Heat shock protein 70 | 29 |
| IPR001245 | Serine-threonine/tyrosine-protein kinase | 50 | IPR001079 | Galectin, carbohydrate recognition domain | 28 |
| IPR019782 | WD40 repeat 2 | 50 | IPR016044 | Filament | 27 |
| IPR000504 | RNA recognition motif domain | 49 | IPR000217 | Tubulin | 26 |
| IPR001757 | ATPase, P-type, K/Mg/Cd/Cu/Zn/Na/Ca/Na/H-transporter | 48 | IPR003008 | Tubulin/FtsZ, GTPase domain | 26 |
| IPR000242 | Protein-tyrosine phosphatase, receptor/non-receptor type | 44 | IPR001322 | Intermediate filament, C-terminal | 25 |
| IPR016040 | NAD(P)-binding domain | 43 | IPR001806 | Ras GTPase | 25 |
| IPR016024 | Armadillo-type fold | 40 | IPR002017 | Spectrin repeat | 25 |
| IPR013753 | Ras | 38 | IPR005225 | Small GTP-binding protein | 25 |
| IPR002110 | Ankyrin repeat | 36 | IPR008280 | Tubulin/FtsZ, C-terminal | 25 |
| IPR013783 | Immunoglobulin-like fold | 36 | IPR013320 | Concanavalin A-like lectin/glucanase, subgroup | 25 |
| IPR008271 | Serine/threonine-protein kinase, active site | 34 | IPR013753 | Ras | 25 |
| IPR012677 | Nucleotide-binding, alpha-beta plait | 34 | IPR017936 | Thioredoxin-like | 25 |
| IPR019775 | WD40 repeat, conserved site | 34 | IPR018316 | Tubulin/FtsZ, 2-layer sandwich domain | 25 |
