# Supplementary material for: Transcriptomic and Proteomic Analyses of a Wolbachia-Free Filarial Parasite Provide Evidence of Trans-Kingdom Horizontal Gene Transfer
Source: PLoS One. 2012 Sep 26;7(9):e45777. doi: 10.1371/journal.pone.0045777 (PMC3458923; doi:10.1371/journal.pone.0045777)
Supplement: Table S4 — KEGG pathway category mappings for Onchocerca flexuosa peptide translations and proteins identified by mass spectroscopy (MS). Peptide translations from 5,159 isogroups and singletons were assigned to 2,049 unique KEGG orthologous (KO) groups, while 608 protein database entries with matches to MS peptides (termed MS proteins) were associated with 446 unique KO groups. The sequences were binned into broad pathway categories based on their association with these KO groups. (DOC) [file pone.0045777.s004.doc]

**Table S4: KEGG pathway category mappings for *O. flexuosa* peptide translations and proteins identified by mass spectroscopy (MS).**

|  | Peptide Translations | | | | MS Proteins | | | |
| --- | --- | --- | --- | --- | --- | --- | --- | --- |
|  | Isogroups & Singletons | | KO Groups | | Proteins | | KO Groups | |
|  | Number | Percent | Number | Percent | Number | Percent | Number | Percent |
| **Metabolism** | **1939** | **37.58%** | **749** | **36.55%** | **278** | **45.72%** | **195** | **43.72%** |
| Carbohydrate Metabolism | 287 | 5.56% | 106 | 5.17% | 101 | 16.61% | 53 | 11.88% |
| Energy Metabolism | 304 | 5.89% | 116 | 5.66% | 86 | 14.14% | 56 | 12.56% |
| Lipid Metabolism | 160 | 3.10% | 70 | 3.42% | 20 | 3.29% | 17 | 3.81% |
| Nucleotide Metabolism | 176 | 3.41% | 76 | 3.71% | 23 | 3.78% | 15 | 3.36% |
| Amino Acid Metabolism | 273 | 5.29% | 93 | 4.54% | 55 | 9.05% | 35 | 7.85% |
| Metabolism of Other Amino Acids | 87 | 1.69% | 34 | 1.66% | 22 | 3.62% | 15 | 3.36% |
| Glycan Biosynthesis and Metabolism | 184 | 3.57% | 75 | 3.66% | 13 | 2.14% | 10 | 2.24% |
| Metabolism of Cofactors and Vitamins | 74 | 1.43% | 44 | 2.15% | 12 | 1.97% | 9 | 2.02% |
| Metabolism of Terpenoids and Polyketides | 46 | 0.89% | 20 | 0.98% | 7 | 1.15% | 4 | 0.90% |
| Biosynthesis of Other Secondary Metabolites | 20 | 0.39% | 9 | 0.44% | 7 | 1.15% | 4 | 0.90% |
| Xenobiotics Biodegradation and Metabolism | 59 | 1.14% | 23 | 1.12% | 14 | 2.30% | 9 | 2.02% |
| Enzyme Families | 631 | 12.23% | 233 | 11.37% | 51 | 8.39% | 39 | 8.74% |
| **Genetic Information Processing** | **2442** | **47.33%** | **1023** | **49.93%** | **233** | **38.32%** | **191** | **42.83%** |
| Transcription | 634 | 12.29% | 283 | 13.81% | 23 | 3.78% | 18 | 4.04% |
| Translation | 493 | 9.56% | 215 | 10.49% | 91 | 14.97% | 77 | 17.26% |
| Folding, Sorting and Degradation | 870 | 16.86% | 350 | 17.08% | 114 | 18.75% | 87 | 19.51% |
| Replication and Repair | 628 | 12.17% | 263 | 12.84% | 25 | 4.11% | 21 | 4.71% |
| **Environmental Information Processing** | **879** | **17.04%** | **320** | **15.62%** | **89** | **14.64%** | **61** | **13.68%** |
| Membrane Transport | 61 | 1.18% | 38 | 1.85% | 6 | 0.99% | 5 | 1.12% |
| Signal Transduction | 526 | 10.20% | 175 | 8.54% | 54 | 8.88% | 32 | 7.17% |
| Signaling Molecules and Interaction | 403 | 7.81% | 142 | 6.93% | 43 | 7.07% | 33 | 7.40% |
| **Cellular Processes** | **1119** | **21.69%** | **379** | **18.50%** | **148** | **24.34%** | **93** | **20.85%** |
| Transport and Catabolism | 368 | 7.13% | 139 | 6.78% | 67 | 11.02% | 46 | 10.31% |
| Cell Motility | 326 | 6.32% | 87 | 4.25% | 64 | 10.53% | 31 | 6.95% |
| Cell Growth and Death | 298 | 5.78% | 93 | 4.54% | 10 | 1.64% | 8 | 1.79% |
| Cell Communication | 288 | 5.58% | 90 | 4.39% | 54 | 8.88% | 28 | 6.28% |
| **Organismal Systems** | **765** | **14.83%** | **252** | **12.30%** | **110** | **18.09%** | **74** | **16.59%** |
| **Human Diseases** | **675** | **13.08%** | **252** | **12.30%** | **147** | **24.18%** | **92** | **20.63%** |
| **Poorly Characterized** | **48** | **0.93%** | **26** | **1.27%** | **2** | **0.33%** | **2** | **0.45%** |
| Total | 5159 |  | 2049 |  | 608 |  | 446 |  |
