# Supplementary material for: Transcriptomic and Proteomic Analyses of a Wolbachia-Free Filarial Parasite Provide Evidence of Trans-Kingdom Horizontal Gene Transfer
Source: PLoS One. 2012 Sep 26;7(9):e45777. doi: 10.1371/journal.pone.0045777 (PMC3458923; doi:10.1371/journal.pone.0045777)
Supplement: Table S5 — KEGG pathway modules represented in Onchocerca flexuosa and Brugia malayi . After being assigned to KEGG orthologous groups, sequences from O. flexuosa (both peptide translations derived from the adult transcriptome and protein database entries with matches to mass spectroscopy peptides) and B. malalyi predicted proteins (WormBase brugpep.WS221.fa) were binned in to KEGG pathway modules in order to compare the metabolic capabilities of the two filarial species. (DOC) [file pone.0045777.s005.doc]

**Table S5: KEGG pathway modules represented in *O. flexuosa* and *B. malayi*.**

| **Module** | **Description** | **Total Enzymes in Module** | ***B. malayi* Proteins** | ***O. flexuosa*** | |
| --- | --- | --- | --- | --- | --- |
|  |  |  |  | **Peptide Translations** | **MS Proteins** |
| M00002 | Glycolysis, core module involving three-carbon compounds | 7 | 6 | 6 | 6 |
| M00003 | Gluconeogenesis, oxaloacetate => fructose-6P | 13 | 8 | 9 | 7 |
| M00004 | Pentose phosphate pathway (Pentose phosphate cycle) | 11 | 8 | 4 | 4 |
| M00005 | PRPP biosynthesis, ribose 5P => PRPP | 1 | 1 | 1 | 0 |
| M00006 | Pentose phosphate pathway, oxidative phase, glucose 6P => ribulose 5P | 6 | 4 | 2 | 3 |
| M00007 | Pentose phosphate pathway, non-oxidative phase, fructose 6P => ribose 5P | 6 | 4 | 2 | 1 |
| M00008 | Entner-Doudoroff pathway, glucose-6P => glyceraldehyde-3P + pyruvate | 6 | 3 | 1 | 2 |
| M00009 | Citrate cycle (TCA cycle, Krebs cycle) | 27 | 12 | 10 | 10 |
| M00010 | Citrate cycle, first carbon oxidation | 5 | 4 | 4 | 3 |
| M00011 | Citrate cycle, second carbon oxidation | 30 | 12 | 10 | 10 |
| M00012 | Glyoxylate cycle | 8 | 4 | 4 | 4 |
| M00013 | Malonate semialdehyde pathway, propanoyl-CoA => Acetyl-CoA | 8 | 5 | 3 | 1 |
| M00014 | Glucuronate pathway (uronate pathway) | 5 | 1 | 1 | 1 |
| M00015 | Proline biosynthesis, glutamate => proline | 4 | 1 | 0 | 0 |
| M00019 | Leucine biosynthesis, pyruvate => 2-oxoisovalerate => leucine | 11 | 1 | 1 | 0 |
| M00020 | Serine biosynthesis, glycerate-3P => serine | 3 | 2 | 1 | 0 |
| M00023 | Tryptophan biosynthesis, chorismate => tryptophan | 14 | 0 | 2 | 0 |
| M00026 | Histidine biosynthesis, PRPP => histidine | 15 | 0 | 1 | 0 |
| M00027 | GABA (gamma-Aminobutyrate) shunt | 6 | 3 | 3 | 1 |
| M00028 | Ornithine biosynthesis, glutamate => ornithine | 15 | 1 | 0 | 0 |
| M00032 | Lysine degradation, lysine => saccharopine => acetoacetyl-CoA | 18 | 5 | 4 | 3 |
| M00034 | Methionine salvage pathway | 17 | 6 | 3 | 2 |
| M00035 | Methionine degradation | 5 | 3 | 2 | 1 |
| M00036 | Leucine degradation, leucine => acetoacetate + acetyl-CoA | 13 | 7 | 4 | 1 |
| M00037 | Melatonin biosynthesis, tryptophan => serotonin => melatonin | 4 | 1 | 1 | 0 |
| M00038 | Tryptophan metabolism, tryptophan => kynurenine => 2-aminomuconate | 9 | 2 | 2 | 0 |
| M00039 | Lignin biosynthesis, cinnamate => lignin | 7 | 1 | 1 | 1 |
| M00042 | Catecholamine biosynthesis, tyrosine => dopamine => noradrenaline => adrenaline | 6 | 2 | 1 | 0 |
| M00043 | Thyroid hormone biosynthesis, tyrosine => triiodothyronine/thyroxine | 1 | 1 | 0 | 0 |
| M00044 | Tyrosine degradation, tyrosine => homogentisate | 2 | 1 | 1 | 0 |
| M00046 | beta-Alanine biosynthesis, cytosine / uracil => beta-alanine | 4 | 1 | 1 | 0 |
| M00047 | Creatine pathway | 3 | 1 | 1 | 0 |
| M00048 | Inosine monophosphate biosynthesis, PRPP + glutamine => IMP | 20 | 1 | 4 | 0 |
| M00049 | Adenine nucleotide biosynthesis, IMP => ADP/dADP,ATP/dATP | 10 | 6 | 5 | 3 |
| M00050 | Guanine nucleotide biosynthesis, IMP => GDP/dGDP,GTP/dGTP | 10 | 6 | 5 | 2 |
| M00051 | Uridine monophosphate biosynthesis, glutamine (+ PRPP) => UMP | 13 | 3 | 5 | 1 |
| M00052 | Pyrimidine ribonucleotide biosynthesis, UMP => UDP/UTP,CDP/CTP | 6 | 3 | 2 | 2 |
| M00053 | Pyrimidine deoxyribonuleotide biosynthesis, CDP/CTP => dCDP/dCTP,dTDP/dTTP | 12 | 6 | 4 | 1 |
| M00055 | N-glycan precursor biosynthesis | 13 | 12 | 8 | 1 |
| M00056 | O-glycan biosynthesis, mucin type core | 7 | 1 | 2 | 1 |
| M00057 | Glycosaminoglycan biosynthesis, linkage tetrasaccharide | 7 | 3 | 3 | 0 |
| M00058 | Glycosaminoglycan biosynthesis, chondroitin sulfate backbone | 5 | 2 | 2 | 0 |
| M00059 | Glycosaminoglycan biosynthesis, heparan sulfate backbone | 10 | 5 | 4 | 0 |
| M00065 | GPI-anchor biosynthesis, core oligosaccharide | 8 | 5 | 2 | 0 |
| M00066 | Lactosylceramide biosynthesis | 2 | 1 | 0 | 0 |
| M00071 | Glycosphingolipid biosynthesis, neolacto-series, LacCer => nLc4Cer | 5 | 1 | 1 | 0 |
| M00072 | Oligosaccharyltransferase | 8 | 5 | 4 | 0 |
| M00073 | N-glycan precursor trimming | 3 | 3 | 2 | 1 |
| M00074 | N-glycan biosynthesis, high-mannose type | 1 | 1 | 0 | 1 |
| M00075 | N-glycan biosynthesis, complex type | 2 | 2 | 2 | 1 |
| M00076 | Dermatan sulfate degradation | 5 | 1 | 1 | 1 |
| M00077 | Chondroitin sulfate degradation | 4 | 1 | 1 | 1 |
| M00078 | Heparan sulfate degradation | 9 | 1 | 1 | 1 |
| M00079 | Keratan sulfate degradation | 4 | 2 | 2 | 1 |
| M00082 | Fatty acid biosynthesis, initiation | 13 | 3 | 2 | 1 |
| M00083 | Fatty acid biosynthesis, elongation | 12 | 2 | 1 | 0 |
| M00085 | Fatty acid biosynthesis, elongation, mitochondria | 6 | 5 | 4 | 3 |
| M00086 | beta-Oxidation, acyl-CoA synthesis | 1 | 1 | 1 | 0 |
| M00087 | beta-Oxidation | 18 | 7 | 6 | 3 |
| M00088 | Ketone body biosynthesis, acetyl-CoA => acetoacetate/3-hydroxybutyrate/acetone | 3 | 1 | 1 | 1 |
| M00089 | Triacylglycerol biosynthesis | 19 | 4 | 4 | 0 |
| M00090 | Phosphatidylcholine (PC) biosynthesis, choline => PC | 5 | 2 | 2 | 0 |
| M00092 | Phosphatidylethanolamine (PE) biosynthesis, ethanolamine => PE | 5 | 4 | 4 | 0 |
| M00093 | Phosphatidylethanolamine (PE) biosynthesis, PA => PS => PE | 3 | 2 | 1 | 0 |
| M00094 | Ceramide biosynthesis | 5 | 3 | 2 | 0 |
| M00095 | C5 isoprenoid biosynthesis, mevalonate pathway | 8 | 6 | 5 | 1 |
| M00096 | C5 isoprenoid biosynthesis, non-mevalonate pathway | 9 | 1 | 1 | 0 |
| M00099 | Sphingosine biosynthesis | 8 | 4 | 2 | 0 |
| M00100 | Sphingosine degradation | 2 | 1 | 1 | 1 |
| M00104 | Bile acid biosynthesis, cholesterol => cholate | 11 | 2 | 2 | 2 |
| M00105 | Bile acid biosynthesis, cholesterol => chenodeoxycholate | 10 | 2 | 2 | 2 |
| M00113 | Jasmonic acid biosynthesis | 8 | 1 | 1 | 1 |
| M00114 | Ascorbate biosynthesis, plants, glucose-6P => ascorbate | 13 | 4 | 4 | 2 |
| M00115 | NAD biosynthesis, aspartate => NAD | 7 | 1 | 0 | 0 |
| M00116 | Menaquinone biosynthesis, chorismate => menaquinone | 11 | 1 | 0 | 0 |
| M00117 | Ubiquinone biosynthesis, prokaryotes, chorismate => ubiquinone | 9 | 1 | 1 | 0 |
| M00118 | Glutathione biosynthesis, glutamate => glutathione | 4 | 3 | 2 | 2 |
| M00119 | Pantothenate biosynthesis, valine/L-aspartate => pantothenate | 3 | 1 | 1 | 0 |
| M00120 | Coenzyme A biosynthesis, pantothenate => CoA | 11 | 2 | 2 | 1 |
| M00121 | Heme biosynthesis, glutamate => protoheme/siroheme | 9 | 3 | 3 | 0 |
| M00124 | Pyridoxal biosynthesis, erythrose-4P => pyridoxal-5P | 6 | 1 | 0 | 0 |
| M00126 | Tetrahydrofolate biosynthesis, GTP => THF | 14 | 3 | 0 | 0 |
| M00128 | Ubiquinone biosynthesis, eukaryotes, chorismate => ubiquinone | 6 | 5 | 4 | 2 |
| M00129 | Ascorbate biosynthesis, animals, glucose-1P => ascorbate | 7 | 3 | 3 | 3 |
| M00130 | Inositol phosphate metabolism, PI=> PIP2 => Ins(1,4,5)P3 => Ins(1,3,4,5)P4 | 11 | 7 | 6 | 1 |
| M00131 | Inositol phosphate metabolism, Ins(1,3,4,5)P4 => Ins(1,3,4)P3 => myo-inositol | 5 | 3 | 2 | 0 |
| M00134 | Polyamine biosynthesis, arginine => ornithine => putrescine | 2 | 1 | 1 | 0 |
| M00135 | GABA biosynthesis, eukaryotes, putrescine => GABA | 5 | 1 | 2 | 1 |
| M00140 | C1-unit interconversion, prokaryotes | 3 | 1 | 1 | 1 |
| M00141 | C1-unit interconversion, eukaryotes | 4 | 1 | 1 | 1 |
| M00142 | Complex I (NADH dehydrogenase), NADH dehydrogenase I | 7 | 1 | 5 | 0 |
| M00143 | Complex I (NADH dehydrogenase), NADH dehydrogenase (ubiquinone) Fe-S protein/flavoprotein | 11 | 10 | 8 | 3 |
| M00144 | Complex I (NADH dehydrogenase), NADH dehydrogenase I | 14 | 1 | 3 | 0 |
| M00146 | Complex I (NADH dehydrogenase), NADH dehydrogenase (ubiquinone) 1 alpha subcomplex | 14 | 9 | 6 | 5 |
| M00147 | Complex I (NADH dehydrogenase), NADH dehydrogenase (ubiquinone) 1 beta subcomplex | 13 | 8 | 7 | 1 |
| M00148 | Complex II (succinate dehydrogenase / fumarate reductase), succinate dehydrogenase (ubiquinone) | 4 | 4 | 4 | 3 |
| M00151 | Complex III (Cytochrome bc1 complex) | 9 | 2 | 2 | 2 |
| M00152 | Complex III (Cytochrome bc1 complex) | 16 | 6 | 5 | 4 |
| M00154 | Complex IV (Cytochrome c oxidase), cytochrome c oxidase | 17 | 9 | 8 | 4 |
| M00155 | Complex IV (Cytochrome c oxidase), cytochrome o ubiquinol oxidase/cytochrome c oxidase/quinol oxidase polypeptide | 13 | 0 | 1 | 0 |
| M00157 | F-type ATPase, bacteria | 8 | 2 | 0 | 0 |
| M00158 | F-type ATPase, eukaryotes | 19 | 10 | 8 | 7 |
| M00160 | V-type ATPase, eukaryotes | 14 | 14 | 11 | 5 |
| M00164 | ATP synthase | 8 | 2 | 0 | 0 |
| M00165 | Reductive pentose phosphate cycle (Calvin cycle) | 16 | 5 | 5 | 3 |
| M00166 | Reductive pentose phosphate cycle, RuBP + CO2 => glyceraldehyde-3P | 5 | 1 | 1 | 1 |
| M00167 | Reductive pentose phosphate cycle, glyceraldehyde-3P => RuBP | 11 | 4 | 4 | 2 |
| M00168 | CAM (Crassulacean acid metabolism), dark | 4 | 2 | 2 | 2 |
| M00169 | CAM (Crassulacean acid metabolism), light | 2 | 1 | 2 | 1 |
| M00170 | C4-dicarboxylic acid cycle, phosphoenolpyruvate carboxykinase type | 6 | 1 | 1 | 1 |
| M00171 | C4-dicarboxylic acid cycle, NAD+ -malic enzyme type | 11 | 3 | 4 | 3 |
| M00172 | C4-dicarboxylic acid cycle, NADP+ -malic enzyme type | 4 | 1 | 2 | 1 |
| M00173 | Reductive citric acid cycle (Arnon-Buchanan cycle) | 29 | 4 | 4 | 3 |
| M00174 | Methane oxidation, methylotroph, methane => CO2 | 19 | 1 | 2 | 1 |
| M00176 | Sulfur reduction, sulfate => H2S | 11 | 1 | 0 | 0 |
| M00177 | Ribosome, eukaryotes | 79 | 78 | 74 | 46 |
| M00178 | Ribosome, bacteria | 55 | 4 | 4 | 0 |
| M00179 | Ribosome, archaea | 67 | 36 | 36 | 18 |
| M00180 | RNA polymerase II, eukaryotes | 12 | 10 | 6 | 0 |
| M00181 | RNA polymerase III, eukaryotes | 16 | 13 | 8 | 0 |
| M00182 | RNA polymerase I, eukaryotes | 14 | 8 | 4 | 0 |
| M00183 | RNA polymerase, bacteria | 6 | 1 | 0 | 0 |
| M00185 | Sulfate transport system | 4 | 0 | 1 | 0 |
| M00190 | Iron(III) transport system | 3 | 0 | 1 | 0 |
| M00223 | Phosphonate transport system | 3 | 0 | 1 | 0 |
| M00236 | Polar amino acid transport system | 3 | 1 | 1 | 0 |
| M00239 | Peptides/nickel transport system | 5 | 0 | 2 | 0 |
| M00248 | Antibiotic transport system | 2 | 0 | 1 | 0 |
| M00260 | DNA polymerase III complex, bacteria | 11 | 2 | 0 | 0 |
| M00261 | DNA polymerase alpha / primase complex | 4 | 4 | 4 | 0 |
| M00262 | DNA polymerase delta complex | 4 | 3 | 2 | 0 |
| M00263 | DNA polymerase epsilon complex | 4 | 3 | 3 | 0 |
| M00284 | Origin recognition complex | 6 | 4 | 4 | 0 |
| M00285 | MCM complex | 6 | 6 | 4 | 0 |
| M00286 | GINS complex | 4 | 4 | 3 | 0 |
| M00288 | RPA complex | 4 | 2 | 2 | 0 |
| M00289 | RF-C complex | 3 | 3 | 3 | 0 |
| M00290 | Holo-TFIIH complex | 10 | 10 | 5 | 0 |
| M00291 | MRN complex | 3 | 2 | 2 | 0 |
| M00292 | MRX complex | 3 | 2 | 2 | 0 |
| M00293 | DNA polymerase zeta complex | 2 | 1 | 1 | 0 |
| M00294 | DNA polymerase gamma complex | 2 | 1 | 1 | 0 |
| M00295 | BRCA1-associated genome surveillance complex (BASC) | 14 | 12 | 11 | 0 |
| M00296 | BER complex | 6 | 3 | 3 | 0 |
| M00297 | DNA-PK complex | 3 | 2 | 2 | 0 |
| M00307 | Pyruvate oxidation, pyruvate => acetyl-CoA | 10 | 4 | 4 | 3 |
| M00331 | Type II general secretion system | 13 | 0 | 1 | 0 |
| M00333 | Type IV secretion system | 12 | 2 | 1 | 0 |
| M00335 | Sec (secretion) system | 13 | 3 | 2 | 0 |
| M00337 | Immunoproteasome | 16 | 11 | 9 | 6 |
| M00338 | Cysteine biosynthesis, homocysteine + serine => cysteine | 3 | 2 | 0 | 0 |
| M00340 | Proteasome, 20S core particle | 14 | 14 | 11 | 8 |
| M00341 | Proteasome, 19S regulatory particle (PA700) | 19 | 18 | 13 | 11 |
| M00343 | Archaeal proteasome | 3 | 0 | 1 | 0 |
| M00344 | Formaldehyde assimilation, xylulose monophosphate pathway | 3 | 2 | 2 | 1 |
| M00345 | Formaldehyde assimilation, ribulose monophosphate pathway | 6 | 1 | 1 | 1 |
| M00346 | Formaldehyde assimilation, serine pathway | 11 | 3 | 2 | 2 |
| M00347 | Methanogenesis, formate => methane | 44 | 0 | 1 | 0 |
| M00350 | Capsaicin biosynthesis, L-Phenylalanine => Capsaicin | 6 | 1 | 0 | 0 |
| M00351 | Spliceosome, U1-snRNP | 10 | 9 | 8 | 4 |
| M00352 | Spliceosome, U2-snRNP | 20 | 20 | 12 | 4 |
| M00353 | Spliceosome, Prp19/CDC5L complex | 9 | 9 | 7 | 1 |
| M00354 | Spliceosome, U4/U6.U5 tri-snRNP | 31 | 30 | 23 | 4 |
| M00355 | Spliceosome, 35S U5-snRNP | 31 | 31 | 24 | 5 |
| M00359 | Aminoacyl-tRNA biosynthesis, eukaryotes | 22 | 22 | 19 | 9 |
| M00360 | Aminoacyl-tRNA biosynthesis, prokaryotes | 31 | 21 | 19 | 9 |
| M00361 | Nucleotide sugar biosynthesis, eukaryotes | 7 | 5 | 5 | 3 |
| M00362 | Nucleotide sugar biosynthesis, prokaryotes | 12 | 5 | 5 | 3 |
| M00364 | C10-C20 isoprenoid biosynthesis, bacteria | 4 | 1 | 1 | 0 |
| M00365 | C10-C20 isoprenoid biosynthesis, archaea | 2 | 1 | 1 | 0 |
| M00366 | C10-C20 isoprenoid biosynthesis, plants | 4 | 2 | 1 | 0 |
| M00367 | C10-C20 isoprenoid biosynthesis, non-plant eukaryotes | 3 | 3 | 2 | 0 |
| M00368 | Ethylene biosynthesis, methionine => ethylene | 3 | 1 | 1 | 0 |
| M00373 | Ethylmalonyl pathway | 12 | 1 | 1 | 1 |
| M00374 | Dicarboxylate-hydroxybutyrate cycle | 30 | 2 | 1 | 2 |
| M00375 | Hydroxypropionate-hydroxybutylate cycle | 21 | 1 | 1 | 1 |
| M00376 | 3-Hydroxypropionate bicycle | 27 | 1 | 2 | 1 |
| M00377 | Reductive acetyl-CoA pathway (Wood-Ljungdahl pathway) | 14 | 1 | 1 | 0 |
