# Supplementary material for: Transcriptomic and Proteomic Analyses of a Wolbachia-Free Filarial Parasite Provide Evidence of Trans-Kingdom Horizontal Gene Transfer
Source: PLoS One. 2012 Sep 26;7(9):e45777. doi: 10.1371/journal.pone.0045777 (PMC3458923; doi:10.1371/journal.pone.0045777)
Supplement: Table S6 — KEGG pathways represented by Wolbachia -like sequences in O. flexuosa . Peptide translations sharing sequence identity with Wolbachia genes were assigned to KEGG orthologous groups and binned into KEGG pathway modules in order to determine what pathways/processes these sequences might be related to. (DOCX) [file pone.0045777.s006.docx]

**Table S6: KEGG pathways represented by *Wolbachia*-like sequences in *O. flexuosa*.**

| **Module** | **Description** | **Total Ezymes in Module** | **Peptide translations** |
| --- | --- | --- | --- |
| M00003 | Gluconeogenesis, oxaloacetate => fructose-6P | 13 | 1 |
| M00004 | Pentose phosphate pathway (Pentose phosphate cycle) | 11 | 2 |
| M00007 | Pentose phosphate pathway, non-oxidative phase, fructose 6P => ribose 5P | 6 | 2 |
| M00009 | Citrate cycle (TCA cycle, Krebs cycle) | 27 | 3 |
| M00010 | Citrate cycle, first carbon oxidation | 5 | 1 |
| M00011 | Citrate cycle, second carbon oxidation | 30 | 2 |
| M00012 | Glyoxylate cycle | 8 | 1 |
| M00036 | Leucine degradation, leucine => acetoacetate + acetyl-CoA | 13 | 1 |
| M00048 | Inosine monophosphate biosynthesis, PRPP + glutamine => IMP | 20 | 1 |
| M00050 | Guanine nucleotide biosynthesis, IMP => GDP/dGDP,GTP/dGTP | 10 | 1 |
| M00051 | Uridine monophosphate biosynthesis, glutamine (+ PRPP) => UMP | 13 | 3 |
| M00121 | Heme biosynthesis, glutamate => protoheme/siroheme | 9 | 1 |
| M00144 | Complex I (NADH dehydrogenase), NADH dehydrogenase I | 14 | 2 |
| M00155 | Complex IV (Cytochrome c oxidase), cytochrome o ubiquinol oxidase/cytochrome c oxidase/quinol oxidase polypeptide | 13 | 1 |
| M00165 | Reductive pentose phosphate cycle (Calvin cycle) | 16 | 2 |
| M00167 | Reductive pentose phosphate cycle, glyceraldehyde-3P => RuBP | 11 | 2 |
| M00173 | Reductive citric acid cycle (Arnon-Buchanan cycle) | 29 | 1 |
| M00178 | Ribosome, bacteria | 55 | 1 |
| M00179 | Ribosome, archaea | 67 | 1 |
| M00190 | Iron(III) transport system | 3 | 1 |
| M00307 | Pyruvate oxidation, pyruvate => acetyl-CoA | 10 | 2 |
| M00333 | Type IV secretion system | 12 | 1 |
| M00359 | Aminoacyl-tRNA biosynthesis, eukaryotes | 22 | 5 |
| M00360 | Aminoacyl-tRNA biosynthesis, prokaryotes | 31 | 6 |
| M00374 | Dicarboxylate-hydroxybutyrate cycle | 30 | 1 |
| M00376 | 3-Hydroxypropionate bicycle | 27 | 1 |
